# The ShGlom*Assay* Combines High-Throughput Drug Screening With Downstream Analyses and Reveals the Protective Role of Vitamin D3 and Calcipotriol on Podocytes

**DOI:** 10.3389/fcell.2022.838086

**Published:** 2022-05-16

**Authors:** Marie-Christin Ristov, Tim Lange, Nadine Artelt, Neetika Nath, Andreas W. Kuss, Jochen Gehrig, Maja Lindenmeyer, Clemens D. Cohen, Sheraz Gul, Karlhans Endlich, Uwe Völker, Nicole Endlich

**Affiliations:** ^1^ Institute of Anatomy and Cell Biology, University Medicine Greifswald, Greifswald, Germany; ^2^ Institute of Bioinformatics, University Medicine Greifswald, Greifswald, Germany; ^3^ Department of Functional Genomics, Interfaculty Institute for Genetics and Functional Genomics, University of Greifswald, Greifswald, Germany; ^4^ Acquifer Imaging GmbH, Heidelberg, Germany; ^5^ DITABIS, Digital Biomedical Imaging Systems AG, Pforzheim, Germany; ^6^ III Department of Medicine, University Medical Center Hamburg-Eppendorf, Hamburg, Germany; ^7^ Nephrological Center, Medical Clinic and Policlinic IV, University of Munich, Munich, Germany; ^8^ Fraunhofer Institute for Translational Medicine and Pharmacology, Fraunhofer Cluster of Excellence Immune-Mediated Diseases CIMD, Hamburg, Germany

**Keywords:** podocyte, CKD—chronic kidney disease, differentiation, glomerulus, screening, compound, vitamin D3

## Abstract

Chronic kidney disease (CKD) is a major public health burden affecting more than 500 million people worldwide. Podocytopathies are the main cause for the majority of CKD cases due to pathogenic morphological as well as molecular biological alterations of postmitotic podocytes. Podocyte de-differentiation is associated with foot process effacement subsequently leading to proteinuria. Since currently no curative drugs are available, high throughput screening methods using a small number of animals are a promising and essential tool to identify potential drugs against CKD in the near future. Our study presents the implementation of the already established mouse Glom*Assay* as a semi-automated high-throughput screening method—shGlom*Assay*—allowing the analysis of several hundreds of FDA-verified compounds in combination with downstream pathway analysis like transcriptomic and proteomic analyses from the same samples, using a small number of animals. In an initial prescreening we have identified vitamin D3 and its analog calcipotriol to be protective on podocytes. Furthermore, by using RT-qPCR, Western blot, and RNA sequencing, we found that mRNA and protein expression of nephrin, the vitamin D receptor and specific podocyte markers were significantly up-regulated due to vitamin D3- and calcipotriol-treatment. In contrast, kidney injury markers were significantly down-regulated. Additionally, we found that vitamin D3 and calcipotriol have had neither influence on the expression of the miR-21 and miR-30a nor on miR-125a/b, a miRNA described to regulate the vitamin D receptor. In summary, we advanced the established mouse Glom*Assay* to a semi-automated high-throughput assay and combined it with downstream analysis techniques by using only a minimum number of animals. Hereby, we identified the vitamin D signaling pathway as podocyte protective and to be counteracting their de-differentiation.

## Introduction

With a prevalence of 10% in the Western world, chronic kidney disease (CKD) is a global public health burden (Bikbov et al., 2020). Diabetes mellitus and arterial hypertension constitute to the main risks for the development of CKD. In case of disease progression or when treated inadequately, it increases the risk for cardiovascular diseases and subsequently leads to the total loss of renal function and end-stage kidney disease (ESKD) (Levey et al., 2007; Collister et al., 2016). In 70% of all CKD cases podocytopathies and the associated impairment of the glomerular filtration barrier (GFB) are causal (Wiggins, 2007). The GFB consists of the fenestrated capillary endothelium, the glomerular basement membrane and the highly differentiated, postmitotic podocytes with their interdigitating foot processes. Adjacent foot processes form cell-cell contacts consisting of specific proteins, including nephrin (NPHS1), forming a size-selective slit diaphragm. Structural changes such as effacement of podocyte foot processes or alterations of slit diaphragm proteins like NPHS1 lead to the loss of the interdigitating pattern between the adjacent podocytes, resulting in proteinuria as an indicator of CKD progression (Bose et al., 2017). In most of these cases, podocyte de-differentiation is the major causal event (May et al., 2014).

Insufficient therapeutic approaches in the treatment of CKD and the underlying podocyte lesions, make dialysis and kidney transplantation inevitable for ESKD patients. This highlights the need for drugs, to expand the current treatment options for CKD patients, which in turn requires appropriate high-throughput screening methods (Feder et al., 2016; Bryer and Susztak, 2018).

Current strategies mostly rely on cell line based- or *in vivo* approaches. Most cell lines represent insufficient podocyte characteristics leading to results hardly translatable to clinical entities. Animal trials negotiate this but require large animal quantities making high-throughput approaches close to impossible (Lee et al., 2015; Bryer and Susztak, 2018).

To address this issue, we recently developed an *in situ* screening model using a transgenic mouse strain that expresses the cyan fluorescent protein (CFP) under the control of a *Nphs1* promoter fragment resulting in cyan fluorescent podocytes when *Nphs1* is expressed. *Nphs1* expression is known to be directly related to podocyte differentiation *in vivo* and spontaneously decreases over time in cultured podocytes (Schiwek et al., 2004; Agarwal et al., 2021). So the Glom*Assay* is using the fluorescence intensity of the isolated glomeruli as a read out for podocyte de-/differentiation over time and in the presence of specific compounds (Kindt et al., 2017). Since novel semi-automated imaging approaches and the related data processing progressed quickly over the last years, imaging based high-throughput screening procedures became more practicable.

In the past, studies have investigated vitamin D3 (vit D) and its complex spectrum of action on various organs, finding potential protective properties of this compound on kidney tissue. Besides its classical working modes as a regulator of calcium and phosphate metabolism, alternative biological signaling pathways of this metabolite have been investigated (Lehmann and Meurer, 2010). Recently, some tissue-protective mechanisms of vit D have been discovered. Even though Wang *et al.* provided experimental data showing that vit D in podocytes contributes to the protection of the kidney against diabetic injuries (Wang et al., 2012), it remains a subject of controversy and therefore further studies are necessary to reveal the role of vit D because it might be an option to treat specific kidney diseases.

Taken togehter, this study shows the application of the Glom*Assay* to a semi-automated high-throughput procedure in combination with downstream analysis techniques. This allows the screening of hundreds of potential drugs and the identification of specific pathways, like the vit D and calcipotriol pathway by using a minimum number of animals.

## Methods

### Transgenic Mice

Transgenic *Nphs1*:CFP-mice (Wong et al., 2000; Cui et al., 2005) were used in the present study. The housing conditions of mice have been described previously (Kindt et al., 2017). Mice at the age of 6 months were used for experiments. All laboratory animal studies have been approved by local authorities (#A3936/3/1) and adhere to the National Institute of Health’s guidelines for the care and use of laboratory animals. All experiments have been carried out in accordance with national animal welfare guidelines (Kilkenny et al., 2010; McGrath and Lilley, 2015).

### Glomeruli Isolation and Culture

Glomeruli were isolated as previously described (Kindt et al., 2017). 96-well plates (Greiner Bio-One GmbH, Frickenhausen, Germany) were coated with collagen IV (Corning, New York, United States) and glomeruli were grown with phenol red-free RPMI-1640 medium supplemented with 10% FBS (both Thermo Fisher Scientific, Waltham, MA, United States) at 37°C and 5% CO^2^. To prevent glomeruli from drying but still ensure gas exchange, we used adhesive Seals (4titude Ltd., Berlin, Germany). Glomerular viability was verified by propidium iodide (Merck KGaA, Darmstadt, Germany) staining performed after manufacturer’s instructions.

### Pharmacological Treatment

The isolated glomeruli were treated with the following substances and final concentrations in the corresponding well throughout all experiments. DMSO (Merck KGaA, 0.1%, dissolved in phenol red-free RPMI-1640 medium with 10% FBS) was used as control-treatment, vit D (100 nM, Santa Cruz Biotechnology, Heidelberg, Germany, dissolved in 0.1% DMSO) and calcipotriol (1 µM MedChemExpress, Monmouth Junction, NJ 08852, United States, dissolved in 0.1% DMSO). All treatments were performed on glomeruli of the same mouse. The vit D concentrations are orientated on the Institute of Medicine (IOM) (Ross et al., 2011).

### Imaging and Fluorescence Quantification

The Acquifer Imaging Machine (IM, DITABIS, Pforzheim, Germany) was used to measure the fluorescence intensity of glomeruli in 96-well plates. In order to estimate the filling density of glomeruli, overview images were taken with a 2x objective without autofocus. Subsequently, four sub-positions per well were determined avoiding overlapping to exclude double-measurements. Sub-positioning is illustrated in Supplementary Figure S1. Afterwards, 16z-slices with a height of 10 µm per sub-position per well were applied. To quantify fluorescence reporter activity within glomeruli, acquired z-stacks were batch maximum projected using a custom written Perl script in combination with Fiji open-source software (available upon request, DITABIS). Maximum projections of one experimental folder were loaded into a z-stack in Fiji. Each z-slice was duplicated and thresholded using the Triangle method. The area, mean intensity, minimum intensity and the integrated intensity were then measured within the masked region of the original image (Supplementary Figure S1). Imaging was performed after 3-, 6- and 9 days past isolation and treatment. These time points have previously been shown to resemble a mild, moderate and strong phenotype, respectively (Kindt et al., 2017).

### RT-PCR

For RNA isolation the glomeruli were cultured for 9 days in 6 well plates (SARSTEDT AG & Co. KG, Nümbrecht, Germany) and treated with DMSO 0.1%, vit D 100 nM and calcipotriol 1 µM. Glomeruli were washed twice with PBS prior to RNA isolation. The RNA isolation was performed with TRI-reagent (Merck KGaA) according to manufacturer’s protocol. The isolated RNA was measured using a photometer (Eppendorf AG, Hamburg, Germany). The Reverse Transcription Kit (Qiagen, Hilden, Germany) was used to synthesize cDNA from equal amounts of denaturated RNA and ranged from 700 ng to 1 µg. Negative controls included no-template and no-reverse-transcriptase controls. RT-PCR was performed on the Mastercycler gradient (Eppendorf AG) by using 10x DreamTaq Green Buffer and DreamTaq DNA Polymerase (both Thermo Fisher Scientific). For RT-qPCR we used iTaq Universal SYBR Green Supermix and Thermal Cycler iQ5 Multicolor Real-Time PCR Detection System (both Bio-Rad Laboratories GmbH, München, Germany). The following primers were used: mouse *Nphs1*, CFP, and Actb as previously described (Kindt et al., 2017); mouse *Vdr*, forward 5´-TCC GGA GAC TCC TCC TCA AA-3´, reverse 5´-AAA AGA CTG GTT GGA GCG CA-3´, 300 bp product size and mouse *Rxra*, forward 5´- CTC AAT GGC GTC CTC AAG GT-3, reverse 5´-AGG CAG TCC TTG TTG TCT CG-3´, 197 bp product size (both Thermo Fisher Scientific). Ct-values were calculated by the Thermal Cycler iQ5 Multicolor Real-Time PCR Detection System with automatically set thresholds and baselines. Raw Ct-values were normalized against ß-actin as endogenous reference gene and the day 0 control by the ΔΔCt-method. Samples with raw Ct-values ≥38 were excluded from the analysis.

### Taqman RT-qPCR

cDNA was synthesized from 10 ng total RNA using Taqman miRNA Assays and the Taqman miRNA Reverse Transcription Kit (Thermo Fisher Scientific). The following Taqman miRNA Assays were used: Hsa-miR-30a-5p: ID #000417; Hsa-miR-21-5p: ID #000397; Hsa-miR-125a-5p: ID #002198; Hsa-miR-125b-5p: ID #000449. RT-reactions were performed after manufacturer’s instructions. Negative controls included no template and no reverse transcriptase controls. The qPCR was performed with the Taqman miRNA Assays described above and the Taqman Universal Master Mix II, no UNG (Thermo Fisher Scientific) following the manufacturer’s instructions. The reaction volumes contained 1.33 μL undiluted cDNA solution and 18.67 μL Master Mix. The qPCR was performed on the Thermal Cycler iQ5 Multicolor Real-Time PCR Detection System with the following cycler scheme: 10 min at 95°C followed by 45 cycles of 15 s at 95°C and 60 s at 60°C. All samples were run in triplicate. Negative controls included the ones from cDNA synthesis and an extra no template control for the qPCR reaction. Standard curves with standard cDNA samples were used for efficiency determination of every single Taqman miRNA Assay. Ct-values were calculated by the Thermal Cycler iQ5 Multicolor Real-Time PCR Detection System with automatically set thresholds and baselines. Raw Ct-values ≥38 were excluded from analysis. All Ct-values were normalized against day 0 controls and against U6 snRNA as endogenous reference gene by the ΔΔCt-method.

### Preparation of Protein Samples

Proteins were isolated from the same samples as the RNA. DNA and protein precipitation were performed using TRI-Reagent (Merck KGaA) according to the manufacturer’s protocol with minor changes: the protein pellet was air-dried and dissolved in 8 M thiourea/2 M urea (Merck KGaA) on a shaker (Eppendorf AG, Hamburg, Germany). Afterwards the dissolved pellet was centrifuged at 10,000 × g for 10 min at 4°C to remove insoluble material. The amount of protein was determined using the Bradford Assay (SERVA Electrophoresis GmbH, Heidelberg, Germany).

### Western Blots

The Western blots with the corresponding antibodies were performed as described before with some changes: TBST (0.1%) and Clarity Western ECL substrates (Bio-Rad Laboratories GmbH) were used. Blots were stripped for usage of alternative antibodies on the same blot (10). The following antibodies were used as previously described (10): guinea pig anti-NPHS1; mouse anti-CFP; rabbit anti-GAPDH; goat anti-guinea pig; goat anti-mouse; goat anti-rabbit; rabbit anti-VDR (1:1000, Cell Signaling Technology, Frankfurt am Main, Germany #12550) and rabbit anti-RXRa (1:500, Cell Signaling Technology #3085). Samples were run in Stain-free TGX gels and quantified with Criterion Stain Free Imager (SFI, Bio-Rad Laboratories GmbH). Whole lane intensities were quantified using Fiji. Specific band intensities were quantified using Fiji and target bands were normalized against GAPDH and whole lane intensities*.* Ratios of vit D-/calcipotriol-treated, normalized intensities against control-treated, normalized intensities were calculated.

### RNA Sequencing

RNA sequencing (RNA_Seq) was performed with glomeruli cultured in 6-well plates for 6 days after treatment with vit D 100 nM and DMSO 0.1%. The RNA isolation was performed as previously described (Kindt et al., 2017). RNA integrity was visualized using an Agilent Bioanalyzer 2100. Before library preparation, 1 µg total RNA was spiked with ERCC (Thermo Fisher Scientific). Subsequently the ribosomal RNA was digested and removed, using a RiboMinus kit (Thermo Fisher Scientific) according to the instructions of the manufacturer. The RNA was then fragmented, barcoded and prepared for sequencing following the TruSeq Stranded Total RNA protocol (Illumina, San Diego, CA, United States). Thus, obtained sequencing libraries were analysed on an Illumina NextSeq machine, using the NextSeq 500/550 High Output Kit v2 (150 cycles, paired end) chemistry (Illumina). For bioinformatic data analysis we performed quality assessments using FASTQC format. This was followed by application of the Trimmomatic package (read trimming tool; (Bolger et al., 2014). With the latter, the following steps were performed: Remove leading low quality or N bases below quality 3 (LEADING:3)—Remove trailing low quality below quality 3 (TRAILING:3)—Scan the read with a 4-base wide sliding window, cutting when the average quality per base drops below 15 (SLIDINGWINDOW:4:15). TopHat (Kim et al., 2013) was used for alignment against the mouse reference genome (mm10). Read counts were determined using R (R Core Team, 2018) DESeq2 (Love et al., 2014) and the transcripts were annotated using AnnotationDbi Bioconductor packages. We thus obtained between 149 × 106 and 168 × 106 reads per sample and the percentage of mapped reads ranged between 94.4 and 95.6 per cent. Gene ontology classification was performed with Panther 16.0 (Mi et al., 2021).

### Microarrays on Human Kidney Biopsies

Biopsies were obtained from patients after informed consent and with approval of the local ethics committees and Affymetrix expression microarrays were performed within the scope of the European Renal cDNA Bank—Kröner-Fresenius Biopsy Bank (Cohen et al., 2002; Martini et al., 2014). Following renal biopsy, the tissue was transferred to RNase inhibitor and micro-dissected into glomeruli and tubulointerstitium. As previously reported (Cohen et al., 2006), total RNA was extracted from micro-dissected glomeruli, followed by reverse transcription and linear amplification. In the present study we used published glomerular expression data of patients with from focal segmental glomerulosclerosis (FSGS, *n* = 23), diabetic nephropathy (DN, *n* = 14) and minimal change disease (MCD, *n* = 14). Kidney biopsies from living donors prior to transplantation (LD, *n* = 42) were used as control (GSE99340, GSE37463, GSE47185, GSE32591). CEL file normalization was performed with the Robust Multichip Average method using RMAExpress (Version 1.0.5) and the human Entrez-Gene custom CDF annotation from Brain Array version 18. The log-transformed dataset was corrected for batch effect using ComBat from the GenePattern pipeline (http://www.broadinstitute.org/cancer/software/genepattern/). To identify differentially expressed genes the SAM (Significance Analysis of Microarrays) method was applied using TiGR (MeV, Version 4.8.1) (Tusher et al., 2001). A q value below 0.05 was considered to be statistically significant.

### Statistics

Statistical analyses were performed by using IBM SPSS Statistics 22.0 (SSPS Inc., Chicago, IL, United States). All data are presented by mean ± SD and statistical significance was determined by one-way ANOVA and *post-hoc* Bonferroni. *p*-values <0.05 were considered as statistically significant.

## Results

### Application of the Glom*Assay* to a Semi-Automated and High-Throughput Assay

To increase the number of compounds to be screened simultaneously, we adapted the Glom*Assay* (Kindt et al., 2017) from a 15-well to a 96-well plate format. This was achieved by the optimization of image acquisition which was converted from manual confocal microscopy to a semi-automated approach using the Imaging Machine (IM) from Acquifer. Here manual z-stack acquisition is replaced by automated, script-based autofocusing followed by 16-slice z-stack acquisition. Furthermore, we also adapted the manual imaging data analysis and evaluation to a semi-automated Fiji-based approach. By using this assay, we could increase the sample and compound number, lower the number of animals needed to 2–3 mice per plate as well as the manual workload and working time to decrease experimental bias.

### Identification of Vit D and Calcipotriol as a Potential Protective Drugs

**FIGURE 1:**
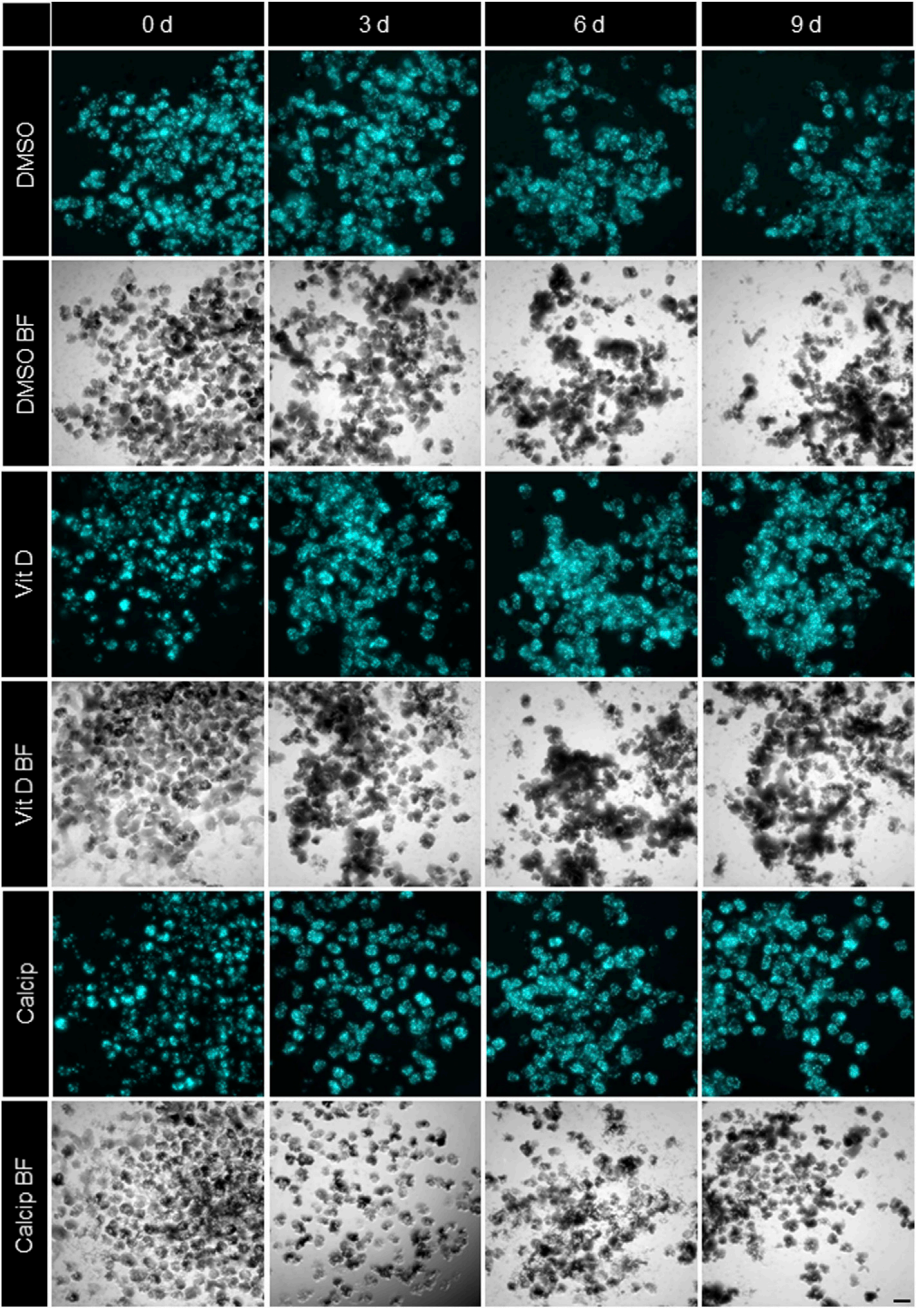
Treatment of isolated glomeruli with vit D and calcipotriol leads to increased CFP fluorescence intensity. Representative Imaging Machine images of cultured glomeruli show different CFP (cyan) fluorescence intensities depending on the treatment. Treatment with vit D 100 nM, 1 µM calcipotriol and 0.1% DMSO. Vit D = vitamin D, calcip = calcipotriol, BF = Brightfield, d = days. Scale bar represents 100 µm. (*n* = 3)

**FIGURE 2:**
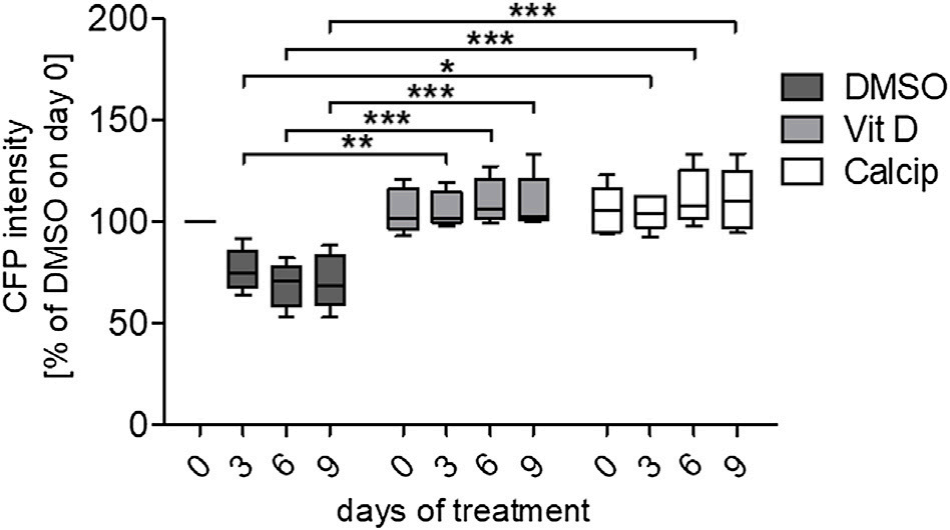
Quantification of the relative CFP intensity after vit D- and calcipotriol-treatment. Vit D- and calcipotriol-treated glomeruli exhibit an enhanced relative CFP intensity compared to DMSO controls. Treatment with vit D 100 nM, 1 µM calcipotriol and 0.1% DMSO. Values presented are medians + Min/Max. *p <0.05; **p <0.01; ***p <0.001. Vit D = vitamin D, calcip = calcipotriol. (*n* = 5)

By using the workflow described before, we measured an increase of the CFP fluorescence after vit D and calcipotriol in an incubation time dependent way. Starting on day 3, the difference in fluorescence intensity between vit D- and DMSO-treated podocytes was 30% (*p* = 0.008). On day 6, this difference increased to 42% (*p* <0.000) and on day 9, CFP fluorescence was 38% more intense for vit D-treated glomeruli compared to DMSO-treatment (*p* <0.001; Figures 1,2). The difference in fluorescence intensity between calcipotriol-treated glomeruli and DMSO was significant and amounts to 29% on day 3 (*p* = 0.015) and 44% on day 6 (*p* <0.001; Figures 1,2). On day 9, CFP fluorescence was 40% more intense after calcipotriol-treatment compared to DMSO-treated samples (*p* <0.000; Figures 1,2).

### Vit D- and Calcipotriol-Treatment Increase the mRNA Expression of *Nphs1* and CFP

**FIGURE 3:**
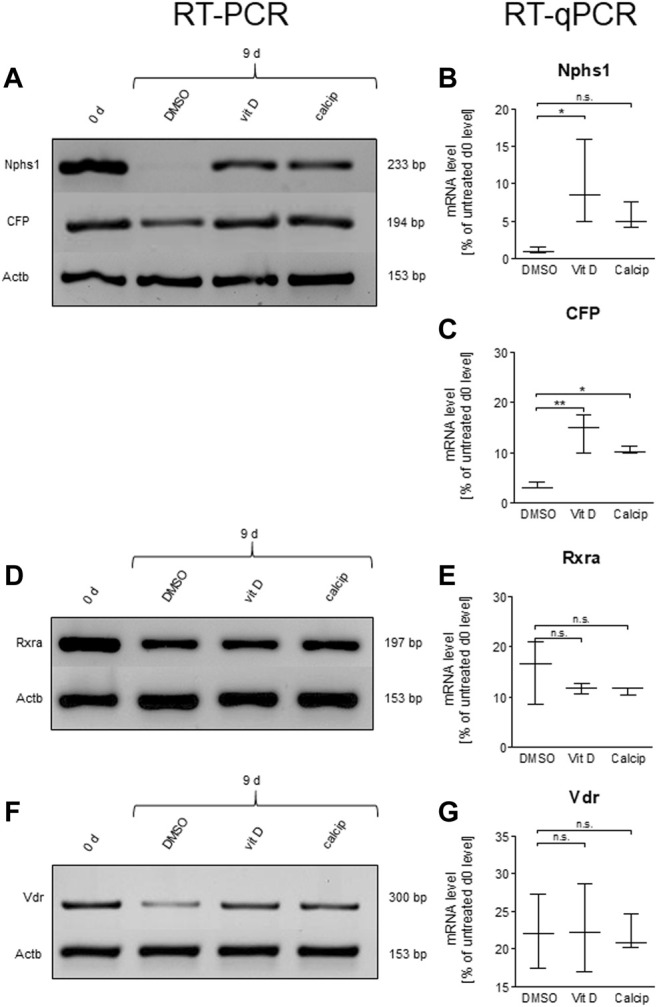
Vit D and calcipotriol increase *Nphs1* and CFP mRNA levels. **(A,D,F)** Representative RT-PCR for *Nphs1*, CFP, *Rxra* and *Vdr* in vit D- and calcipotriol-treated mouse glomeruli after 9 days and untreated glomeruli on day 0. **(B,C,E,G)** Quantification of mRNA levels by RT-qPCR. The mRNA levels of *Nphs1* and CFP are significantly increased by the treatment with vit D (100 nM) and calcipotriol (1 µM) compared to DMSO (0.1%). Values are presented as medians + Min/Max. *p <0.05; **p <0.01; ***p <0.001. Vit D = vitamin D, calcip = calcipotriol. (*n* = 3).

To verify the effect of vit D and calcipotriol obtained by IM, we determined the mRNA levels by RT-qPCR (Figures 3B,C,E,G) and by RT-PCR (Figures 3A,D,F).

By treating the podocytes with vit D, the mRNA levels of *Nphs1* were significantly increased compared to the DMSO control (*p* = 0.039). The treatment with calcipotriol also showed increased expression levels, but these were not in the significant range (*p* = 0.575; Figures 3A,B).

The mRNA levels of CFP were significantly increased compared to DMSO in both vit D- (*p* = 0.001) and calcipotriol-treatment (*p* = 0.012; Figures 3A,C).

In order to obtain further information on the underlying signaling pathway, we also determined the mRNA levels of *Vdr* and its nuclear interaction partner *Rxra* by RT-qPCR (Figures 3D–G). Both receptors showed no significant differences on the mRNA level between vit D-, calcipotriol- and DMSO-treated glomeruli (Figures 3E,G).

### Increased Expression Levels of VDR After Treatment of Glomeruli With Vit D or Calcipotriol

**FIGURE 4:**
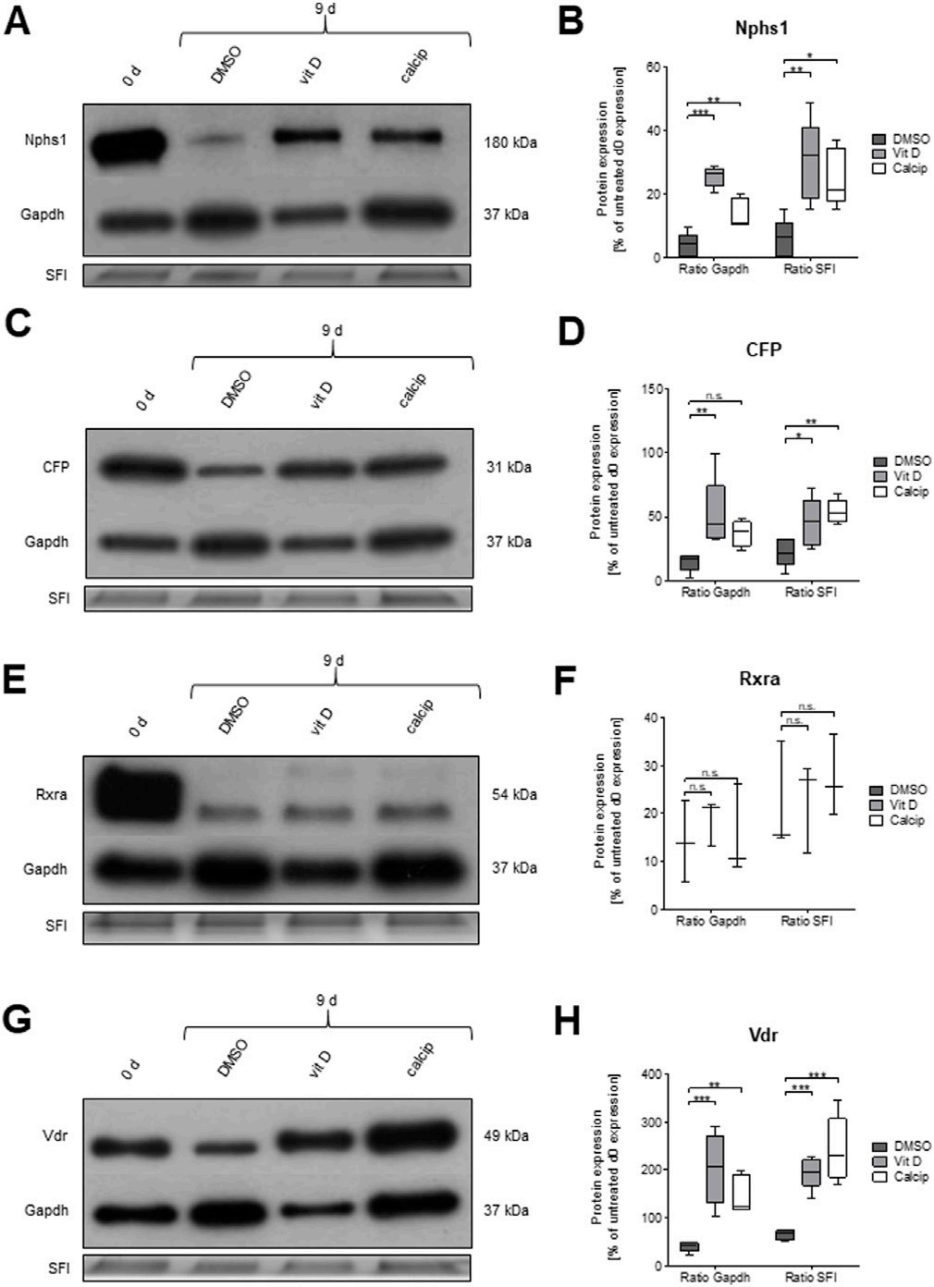
Treatment of glomeruli with vit D and calcipotriol leads to increased protein levels of NPHS1, CFP and VDR after 9 days. **(A,C,E,G)** Representative Western blots for NPHS1, CFP, RXRa, and VDR in vit D- and calcipotriol-treated mouse glomeruli after 9 days and untreated glomeruli on day 0. **(B,D,F,H)** Quantification of Western blot lanes. The protein expression of NPHS1, CFP, and VDR is significantly increased after treatment with vit D and calcipotriol compared to DMSO. Treatment was done with 100 nM vit D, 1 µM calcipotriol, and 0.1% DMSO. Values are presented as medians + Min/Max. *p <0.05; **p <0.01; ***p <0.001. Vit D = vitamin D, calcip = calcipotriol, SFI = Stain Free Imager. (*n* = 5, 5, 3, 5).

To verify the effect of vit D and calcipotriol on the protein level, we also applied Western blot analysis. Data were normalized against GAPDH and against the total protein load. Both normalization methods are shown in Figures 4A–H.

The protein expression of CFP was significantly increased after the treatment with vit D compared to DMSO (GAPDH *p* = 0.007; SFI *p* = 0.043), regardless of the normalization method (Figures 4C,D).

A similar effect was observed for NPHS1 (GAPDH *p* <0.001; SFI *p* = 0.002; Figures 4A,B) by the treatment of glomeruli with vit D. A treatment of glomeruli with calcipotriol resulted in an increase of CFP expression compared to DMSO, although this effect was only significant when normalized with SFI (*p* = 0.004; Figure 4D). NPHS1 showed a significant increase in protein expression by calcipotriol compared to DMSO, regardless of the normalization method (GAPDH *p* = 0.002; SFI *p* = 0.013; Figure 4B).

Protein expression levels of RXRa were similar to the mRNA levels. There were no significant differences in the protein levels between vit D and DMSO and between calcipotriol and DMSO independent of the normalization method (Figures 4E,F).

On the other hand, protein levels and mRNA levels differed strongly regarding *Vdr*. The protein expression of VDR was significantly increased after the treatment with vit D (GAPDH *p* <0.001; SFI *p* <0.001) and calcipotriol (GAPDH *p* = 0.006; SFI *p* <0.001) compared to DMSO, regardless of normalization (Figures 4G,H). Furthermore, VDR was the only of the 4 tested targets where the protein expression was significantly increased after 9 days of treatment with vit D and calcipotriol compared to day 0 (Figure 4H). The significance of this effect was independent of the chosen normalization method for vit D-treatment (GAPDH *p* = 0.009; SFI *p* = 0.007) and depended on the chosen normalization method for the treatment with calcipotriol (GAPDH *p* = 0.573; SFI *p* <0.001; Figure 4H).

### Regulated Expression Levels of miRNA-30a, -21, -125a, -125b

**FIGURE 5:**
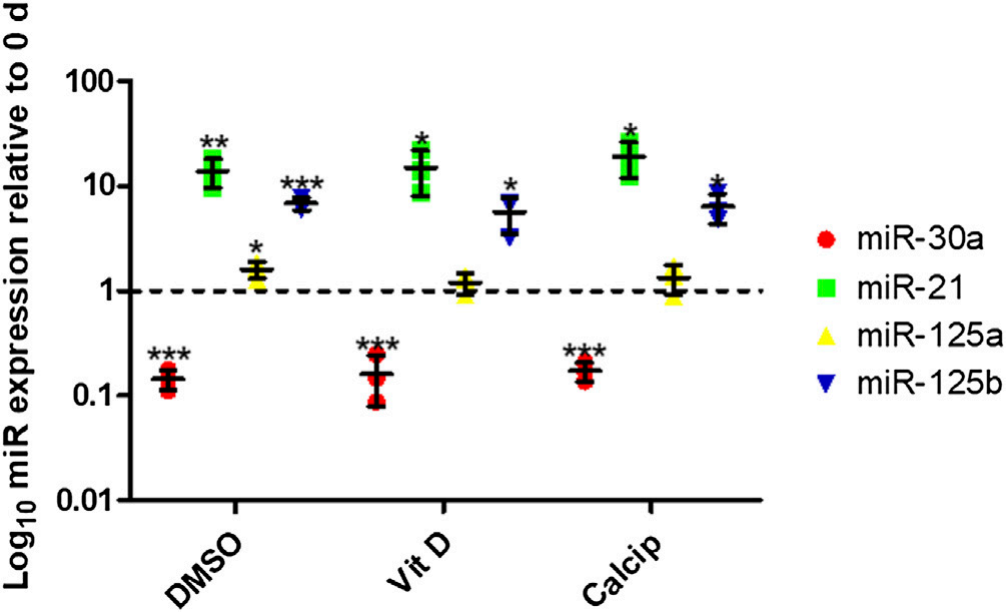
Treatment with vit D and calcipotriol has no effect on specific miR-expression after de-differentiation. After 9 days de-differentiation, miR-30a is down-regulated, while miR-21, miR-125a, and miR-125b are up-regulated. Vit D and calcipotriol showed no significant effect. Treatment with 100 nM vit D, 1 µM calcipotriol, and 0.1% DMSO. Values are presented as log_10_ mean expression (±SD) of each miRNA relative to day 0-controls. *p <0.05; **p <0.01; ***p <0.001. Vit D = vitamin D, calcip = calcipotriol, 0 days = day 0. (*n* = 3)

To analyze the expression of miR-30a, -21, -125a, -125b in podocyte de-differentiation and to observe the effect of vit D on the expression of these miRs, we measured their expression levels in freshly isolated (day 0) and 9 days cultured glomeruli that were treated with vit D, calcipotriol as well as DMSO (control) by RT-qPCR. MiR-21 was up-regulated after 9 days cultivation under control (13.9-fold, *p* = 0.006), vit D- (14.9-fold, *p* = 0.024) and calcipotriol-treatment (19.2-fold, *p* = 0.011). MiR-30a was down-regulated after 9 days cultivation under control conditions (0.1-fold, *p* <0.001), vit D (0.2-fold, *p* <0.001) and calcipotriol (0.2-fold, *p* <0.001). In contrast, miR-125a was up-regulated after the treatment with DMSO (1.6-fold, *p* = 0.025), vit D (1.2-fold, *p* = 0.257) and calcipotriol (1.3-fold, *p* = 0.244) for 9 days. MiR-125b was also up-regulated after a treatment with DMSO (6.8-fold, *p* <0.000), vit D and calcipotriol (5.6-fold, *p* = 0.018; 6.4-fold, *p* = 0.010, respectively) for 9 days. We could not detect any significant differences between the treatment groups at 9 days (Figure 5).

### RNA_Seq Reveals an Up-Regulation of Podocyte-Specific Genes

**FIGURE 6:**
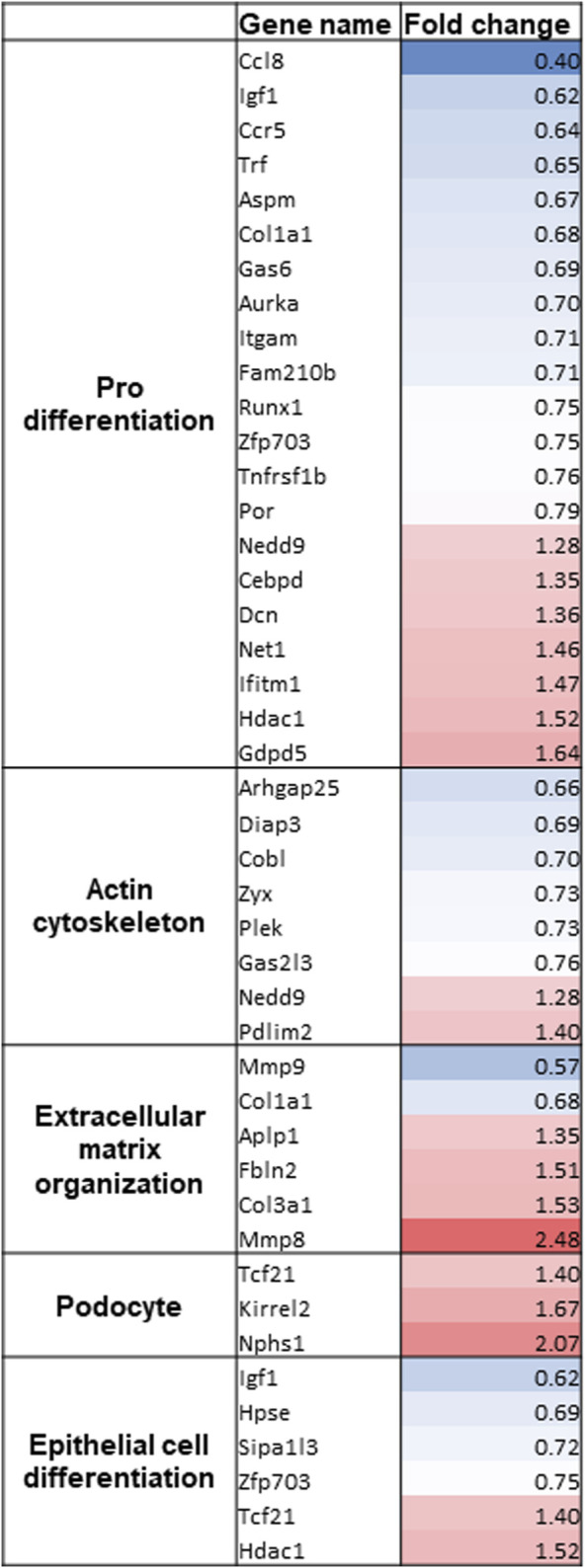
Gene clustering of the most regulated genes after RNA_Seq of vit D-treated glomeruli. Gene names and fold change of significantly regulated genes are shown. Gene clustering was performed using Panther 16.0. Treatment with vit D 100 nM and 0.1% DMSO, respectively. Red = significantly up-regulated genes, blue = significantly down-regulated genes.

As no differences between vit D- and calcipotriol-treated glomeruli could be detected, we performed RNA_Seq only with vit D-treated glomeruli after 6 days in culture. The raw reads were normalized against the geometric mean and normalized against DMSO-treatment at 6 days. After Benjamini–Hochberg correction, we obtained 113 significantly differentially expressed genes. Data distribution is displayed as Volcano plot and MA plot (Supplementary Figure S2). We identified 45 significantly up-regulated and 68 significantly down-regulated genes. Amongst the most up-regulated genes, podocyte-specific markers like *Nphs1*, *Kirrel2* and *Tcf21* were found. We could also find genes involved in epithelial cell differentiation, actin cytoskeleton and extracellular matrix organization (Figure 6). Furthermore, we identified disease markers such as *CD163* and *Mmp9* as well as inflammatory and immune cell markers such as *Gatm*, *Clec10a*, *Stab1,* and *Fcna* as the most down-regulated genes. All significantly regulated genes are shown in Supplementary Table S1.

### Microarrays on Human Kidney Biopsies

**FIGURE 7:**
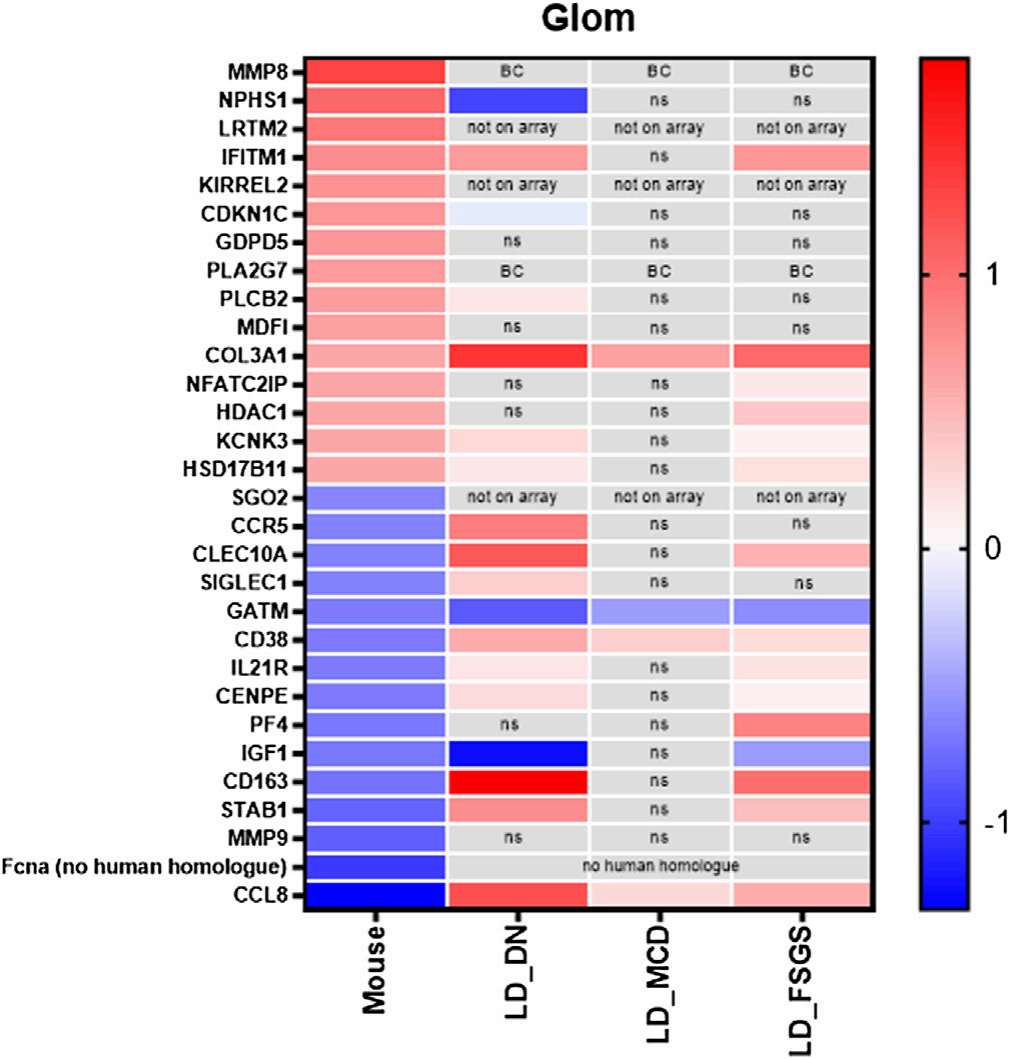
Compilation of the 15 most up-regulated and down-regulated genes and their expression in patients with FSGS, DN and MCD compared to living donors. Data are represented as log fold change, a q-value <5% was considered as significant. Red: up-regulated compared to controls; blue: down-regulated compared to controls. ns: not significant; BC: below cutoff, LD = living donors.

Next, we tested for gene expression alteration in glomerular disease for genes found to be regulated by treatment with vit D and calcipotriol. Microarray data of micro-dissected glomeruli of biopsies of patients with FSGS, DN, and MCD were analyzed. Genes known to be up-regulated by the treatment with vit D and calcipotriol in the shGlom*Assay* were found to be down-regulated in disease. For example, the slit diaphragm protein *Nphs1* and the cycline-dependent kinase inhibitor *Cdkn1c* are down-regulated in DN patients in contrast to the vit D-treated glomeruli. On the other hand, *Clec10a*, *ll21r*, *Cenpe*, *Cd163*, *Stab1,* and *Ccl8* which became down-regulated by vit D and calcipotriol, were up-regulated in FSGS and DN patients. Furthermore, the same regulation was found for the cytokine *Ccl8* (in MCD), *Siglec1* (in DN), *Cd38* (in DN-, MCD and FSGS) and *Pf4* (in FSGS) all of which were up-regulated in contrast to the vit D- and calcipotriol-treated glomeruli. We also identified genes that showed the same regulation direction compared to vit D-treated glomeruli like *Ifitm6*, *Kcnk3,* and *Hsd17b11* that were up-regulated in patients with DN and FSGS. *Plcb2* is up-regulated in DN patients only, whereas *Col3a* is up-regulated in all investigated glomerulopathies. *Nfatc2ip* and *Hdac1* are only up-regulated in FSGS patients. In contrast to this, there are only *Gatm* and *Igf1* that showed the same regulation as the vit D-treated glomeruli in patients with from FSGS, MCD, and DN and in FSGS and DN patients, respectively (Figure 7, Supplementary Table S1).

## Discussion

Glomerulopathies are the main cause for the development of CKD. De-differentiation of podocytes is the leading mechanism and plays a key role in the development of various renal diseases such as diabetic glomerulopathy. Until today, there are no healing or protective drugs available. Therefore, great efforts must be made to identify chemical compounds and their signaling pathways that can stop and reverse this de-differentiation process, making screening models indispensable.

Our group has developed a screening model, the Glom*Assay*, using isolated glomeruli from transgenic *Nphs1*:CFP-mice to study the effect of pharmaceutical substances on podocyte differentiation *in situ*. This assay can be used to evaluate the effect of compounds on the interaction between podocytes, endothelial cells, and mesangial cells, resembling the *in vivo* situation better than permanent podocyte cell lines (Kindt et al., 2017).

Since we wanted to increase the number of drugs that could be screened simultaneously, we further developed the Glom*Assa*y by the use of a semi-automated imaging machine. To address this, we have established a semi-quantitative and high-throughput screening platform (shGlom*Assay*). The shGlom*Assay* screening platform bridges the gap between the use of immortalized podocyte cell lines (Yamauchi et al., 2006) and whole animal experiments. This is a major step forward because, on the one hand, currently available podocyte cell lines are hardly comparable with podocytes *in vivo* and *in situ*. The expression of podocyte-specific proteins like TCF21 and NPHS1 is either completely lost or severely reduced in theses cell lines, the morphology of these podocytes is completely different to *in vivo* podocytes and the matrix composition, which has an important influence on podocyte differentiation, is not comparable (Agarwal et al., 2021). On the other hand, especially rodent models require a high number of animals, aggravating applicability due to the high time requirements, animal right restrictions, ethical issues and readout systems.

In contrast to this, the shGlom*Assay* allows the analysis of a huge variety of different compounds on podocyte differentiation by combining the advantages of *in vitro* and *in vivo* models (Kindt et al., 2017).

Our screening by the shGlom*Assay* identified a podocyte protective effect of vit D, a compound whose effect on podocytes has been controversially discussed in the past. Besides its role in calcium and phosphate metabolism, renoprotective properties of vit D have been found in previous work (Wang et al., 2012). However, high vit D levels are also thought to be associated with severe kidney damage and even kidney failure, which is often related to genetic polymorphisms (Tripathi et al., 2010; Kramer, 2015; Wani et al., 2016).

By using the *s*hGlom*Assay*, we observed that the podocyte-specific CFP fluorescence, driven by the *Nphs1* promoter, is significantly increased after treatment with vit D as well as its analogue calcipotriol compared to controls. This indicates that vit D as well as calcipotriol strongly up-regulate the *Nphs1* expression of *in situ* podocytes. This is in agreement with the observation of Shi et al. showing that the treatment of spontaneously hypertensive rats with vit D resulted in a decrease of proteinuria, increase of serum albumin, as well as a reduction of podocyte injury compared to untreated rats (Shi et al., 2018). Here in our shGlom*Assay*, we could show that the mRNA and protein levels of CFP and *Nphs1* increased simultaneously by the treatment with vit D and calcipotriol. Although *Nphs1* mRNA levels were not significantly increased after calcipotriol-treatment, they showed a clear trend towards up-regulation. This was also confirmed by the significantly increased protein levels in Western blot analysis. In a diabetic rat model, it was demonstrated that the glucose-mediated down-regulation of *Nphs1* could be ameliorated by treatment with a vit D analogue (Trohatou et al., 2017). We could show that the difference in CFP expression reflects NPHS1 expression making the shGlom*Assay* an ideal model for drug screening. It was further shown in animal models as well as in patients that NPHS1 is an essential slit diaphragm protein which is compromised during de-differentiation. A sufficient expression of this specific protein is necessary to maintain the complex podocyte morphology and function of the GFB (Patrakka and Tryggvason, 2007). Putaala and coworkers generated *Nphs1*-deficient mice which died within 24 h after birth and they showed electron microscopically effaced podocytes (Putaala et al., 2001). Gene mutations of *Nphs1* are also associated with missing slit diaphragms and proteinuria (Patrakka et al., 2000). Since impaired *Nphs1* expression is closely related to the development of CKD, it was important to further investigate the regulatory mechanisms of this protein. Deb and coworkers have already demonstrated that vit D influences *Nphs1* expression by acting on a vit D response element in the proximal *Nphs1* promoter (Deb et al., 2011). This effect may indicate that the de-differentiation of vit D- and calcipotriol-treated podocytes progresses more slowly as compared to controls. This is important and supported by the RNA_Seq data of the glomeruli in the shGlom*Assay* showing an up-regulation of podocyte genes like *Nphs1*, *Kirrel2*, *Tcf21,* and *Mmp8* and a down-regulation of genes that are involved in inflammation and fibrosis after vit D-treatment (Supplementary Table S1). Additionally, we found that vit D-treated glomeruli showed adverse regulation of genes that are involved in kidney diseases which was revealed by microarrays of FSGS, DN, and MCD patients (Supplementary Table S1).

Since it is published that vit D binds to the *Vdr* and the *Vdr*/*Rxra* heterodimer, we studied the expression of these receptors under our shGlom*Assay* conditions (Christakos et al., 2016). We observed a significant increase of *Vdr* due to the treatment of vit D as well as calcipotriol, which is in agreement with the results received from spontaneously hypertensive rats (Shi et al., 2018).

While *Vdr* is regulated by the treatment with vit D and calcipotriol, surprisingly, no changes in the *Rxra* expression was observed. This suggested an involvement of another unknown protein in the vit D signaling pathway. Since Okamura and colleagues have shown that vit D-induced *Nphs1* expression can be the result of the interaction of the retinoic acid receptor (*Rara*) and *Vdr* in a *Rxra*-independent way (Okamura et al., 2009), we explored this in our study. However, RNA_Seq data have shown that there is no significant regulation for both receptors.

Recently, miRNAs have been shown to play a pivotal role in podocyte homeostasis and in vit D signaling which underlies a complex regulatory network. In the present study, we selected two miRNAs known as typical kidney injury biomarkers. First of all, miR-30a is known to be down-regulated in de-differentiated podocytes and in kidney diseases (Wu et al., 2014). Here in our shGlom*Assay*, we also found a significant down-regulation of miR-30a after 9 days. In contrast, miR-21, described to be a kidney injury marker (Peters et al., 2020) and up-regulated in urinary exosomes of kidney patients (Lange et al., 2019), was also significantly up-regulation in the shGlom*Assay*.

Other miRs, like miR-125a and miR-125b which are known to target *Vdr* (Zenata and Vrzal, 2017), were found to be regulated in our model. Our experiments show that miR-125b, which seems to have a higher impact on *Vdr* translation than miR-125a (Zenata and Vrzal, 2017), was up-regulated in our shGlom*Assay* already under control conditions. However, vit D-treatment had no effect on the expression of miR-30a, miR-21, miR-125a and miR-125b which was surprising because miR-125b has previously been described to regulate the *Vdr* as well as Cyp24a1, a vit D inactivator (40) and is itself regulated by vit D (Giangreco et al., 2013).

In summary, we have shown that our previously described Glom*Assay* can be adapted to a high throughput compound screening model identifying compounds that are important for podocyte differentiation. We also confirmed the protective effect of vit D and calcipotriol in our model and identified new target genes involved in this signaling pathway.

## Data Availability

The datasets presented in this study can be found in online repositories. The names of the repository/repositories and accession number(s) can be found below: Zenodo; doi: 10.5281/zenodo.6358568.
