# Supplementary material for: The ShGlomAssay Combines High-Throughput Drug Screening With Downstream Analyses and Reveals the Protective Role of Vitamin D3 and Calcipotriol on Podocytes
Source: Front Cell Dev Biol. 2022 May 16;10:838086. doi: 10.3389/fcell.2022.838086 (PMC9150175; doi:10.3389/fcell.2022.838086)
Supplement: Supplementary file 1 [file Table1.pdf]

Supplementary Table 1. Compilation of the 15 most up-regulated and down-regulated genes with kidney disease-relevant publications and their expression in patients with FSGS, DN and MCD. Direc; Direction of gene regulation, Red; up-regulation, green; down-regulation, gray; no data.

	Direc	References	Results	LD_DN		LD_MCD		LD_FSGS	
				Log Fold Change	q-value (%)	Log Fold Change	q-value (%)	Log Fold Change	q-value (%)
**Mmp8**	↑	DOI: 10.1677/JOE-08-0241	Vit D treatment increases Mmp8						
		DOI: 10.1053/j.gastro.2003.12.045	Mmp8 treatment reduces liver cirrhosis						
		DOI: 10.3109/0886022X.2014.996842	Mmp8 null mice has more severe kidney damage than Mmp8 wild-type						
**Nphs1**	↑	DOI: 10.1038/sj.ki.5001625	Up-regulation of nephrin promoter activity and mRNA by vit D	-0.96	0.00	0.07	NaN	-0.08	12.70
		DOI: 10.1093/ndt/gfp243	Up-regulation of nephrin promoter activity and mRNA by vit D						
		DOI: 10.1074/jbc.M111.269118	Up-regulation of nephrin promoter activity and mRNA by vit D						
		PMCID: PMC7447091	Higher Nphs1 expression and reduced kidney injury after calcitriol treatment						
**Lrtm2**	↑								
**Ifitm6**	↑			0.68	0.00	0.07	49.43	0.70	0.00
**Kirrel2**	↑	DOI: 10.1042/CS20130258	Down-regulation of Kirrel2 in glomerular kidney diseases						
		DOI: 10.1093/ndt/gfr576	Kirrel2 is regulated by NF-κB and NF-κB is regulated by vit D						
		DOI: 10.1016/j.ejphar.2018.04.032	Kirrel2 is regulated by NF-κB and NF-κB is regulated by vit D						
**Cdkn1c**	↑	DOI: 10.1159/000101793	Role of Cdkn1c in podocyte damage	-0.10	4.01	-0.15	27.70	-0.16	5.07
		DOI: 10.1158/1535-7163.MCT-07-2222	Up-regulation of Cdkn1c by vit D analogue						
**Gdpd5**	↑	DOI: 10.1073/pnas.0805496105	Inhibition of Gdpd5 increases osmo-protective glycerol phosphate	0.10	9.13	-0.02	NaN	0.08	9.68
**Pla2g7**	↑	DOI: 10.1016/j.amjms.2016.07.002	Lp-PLA2 activity increases in patients with CKD						
		DOI: 10.1007/s40620-018-0521-3	PLA2 activity is increased in dialysis patients						
**Plcb2**	↑	DOI: 10.1074/jbc.272.18.11902	Plcb2 involved in vit D dependent calcium mobilization	0.17	0.90	0.02	47.71	-0.07	14.55
**Mdfi**	↑	DOI: 10.1016/s0167-4889(01)00099-4	Vit D enhances the expression of I-mfa	-0.04	25.31	0.02	47.71	-0.10	6.14
		DOI: 10.1152/ajprenal.00508.2019	L-mfa increases fibronectin expression						
**Col3a1**	↑	DOI: 10.1590/1414-431X20198333	Col3a1 is significantly correlated with CKD	1.40	0.00	0.65	0.74	1.04	0.00
		DOI: 10.1016/j.pupt.2019.01.003	Reduction of pulmonary fibrosis through Col3a1 decrease by vit D						
**Nfatc2ip**	↑			0.03	27.22	-0.02	NaN	0.14	0.09
**Hdac1**	↑	DOI: 10.1172/JCI124030	Inhibition of Hdac1 leads to improvement of proteinuria and glomerulosclerosis	0.14	5.27	0.18	5.58	0.39	0.00
		DOI: 10.1016/j.jmb.2015.07.011	Hdac1 leads to Vdr down-regulation						
**Kcnk3**	↑	DOI: 10.1152/ajplung.00475.2019	Vit D significantly increases Kcnk3 mRNA in human smooth muscle cell	0.25	0.07	0.07	20.95	0.08	2.51
**Hsd17b11**	↑			0.16	4.62	-0.03	NaN	0.21	0.92
**Sgol2**	↓	DOI: 10.1681/ASN.2009040379	Sgol2 is up-regulated after podocin inactivation in isolated mouse glomeruli						
		DOI: 10.1681/ASN.2010080860	Sgol2 is up-regulated in Wt-1 mutant mice						
**Ccrf5**	↓	DOI: 10.1681/ASN.2008040432	Ccrf5 is inflammation associated in dialysis patients						
**Clec10a**	↓	DOI: 10.1038/ki.2011.217	Clec10a as a marker for pro-inflammatory tissue macrophages in CKD	1.16	0.00	0.12	16.27	0.53	0.00
**Siglec1**	↓	DOI: 10.1093/ndt/gfaa121	Siglec1 is up-regulated in tubular interstitium in diabetic nephropathy	0.33	0.00	0.07	24.11	0.03	NaN
		DOI: 10.1016/j.berh.2019.06.004	Siglec1 as a biomarker for Lupus nephritis						
**Gatm**	↓	DOI: 10.1681/ASN.2017111179	Gatm aggregates trigger inflammasome in kidney failure	-0.85	0.00	-0.50	0.31	-0.58	0.00
**Cd38**	↓	DOI: 10.1111/j.1582-4934.2011.01462.x	Anti- epithelial-to-mesenchymal transition	0.56	0.00	0.32	0.00	0.23	0.04
**Il21r**	↓	DOI: 10.4049/jimmunol.1601687	Il21r blockade ameliorates diabetic nephropathy	0.17	0.30	0.08	18.05	0.19	0.02
**Cenpe**	↓	DOI: 10.1038/labinvest.2010.188	Cenpe is up-regulated under high glucose	0.23	1.63	0.16	25.64	0.10	3.04
**Pf4**	↓	DOI: 10.1177/1076029617729216	Pf4 is up-regulated in dialysis patients with heart failure	-0.24	17.83	0.56	23.31	0.86	0.00
**Igf1**	↓	DOI: 10.15252/emmm.201404916	Igf1 inhibition prevents mitochondria related kidney failure	-1.25	0.00	-0.52	12.05	-0.52	0.55
**Cd163**	↓	DOI: 10.1681/ASN.2019121285	Cd163 as a lupus nephritis-urine biomarker	1.79	0.00	-0.41	43.94	0.98	0.30
		DOI: 10.1210/en.2014-1020	Cd163 is upregulated in STZ-rats after vit D treatment						
**Stab1**	↓	DOI: 10.1038/nrendo.2010.18	Stab1 as an alternatively activated macrophage marker	0.78	0.00	0.12	14.89	0.45	0.00
**Mmp9**	↓	DOI: 10.1038/ki.2014.67	Mmp9 is up-regulated in diabetic nephropathy	0.06	25.31	-0.43	19.75	-0.06	20.40
**Fcna**	↓	DOI: 10.1038/s41598-018-31887-4	Fcna is up-regulated in monocyte macrophages						
**Ccl8**	↓	DOI: 10.1016/j.kint.2020.05.052	Ccl8 is senescence associated	1.20	0.00	0.25	1.30	0.56	0.00
