# Supplementary figures and images for: The ShGlomAssay Combines High-Throughput Drug Screening With Downstream Analyses and Reveals the Protective Role of Vitamin D3 and Calcipotriol on Podocytes
Source: Front Cell Dev Biol. 2022 May 16;10:838086. doi: 10.3389/fcell.2022.838086 (PMC9150175; doi:10.3389/fcell.2022.838086)

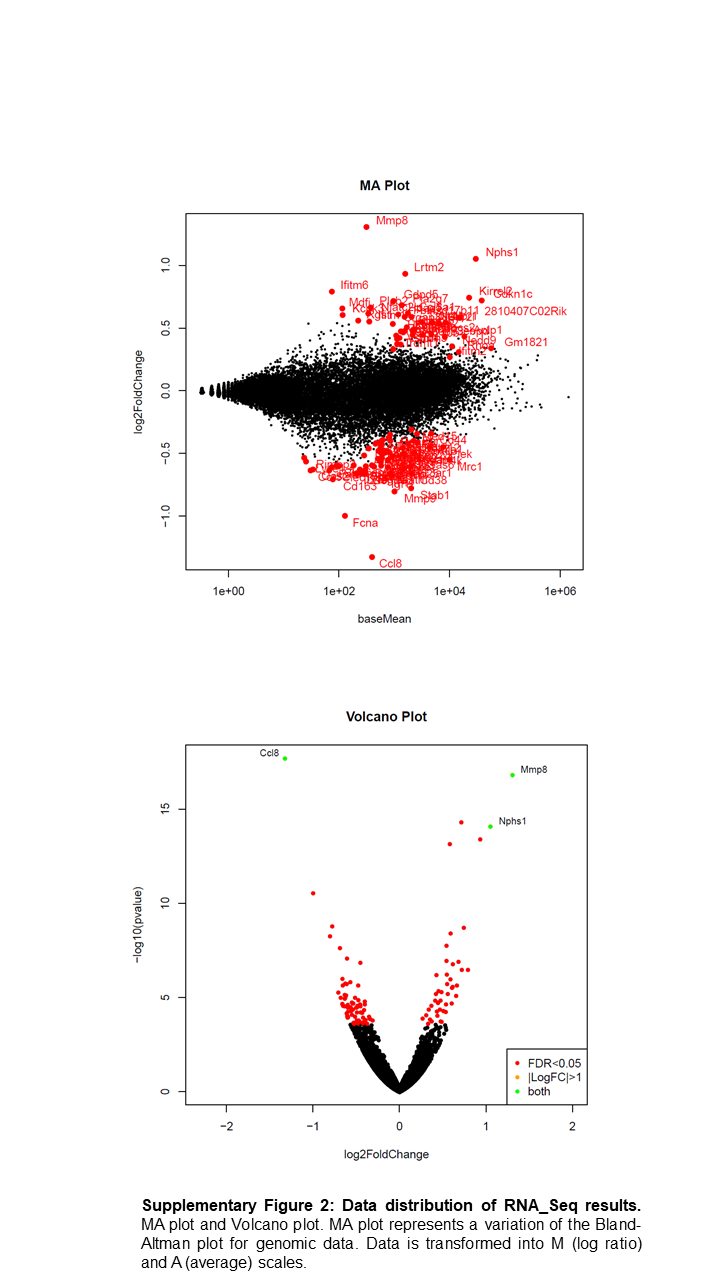

Supplement: Supplementary file 2 [file Image2.tif]

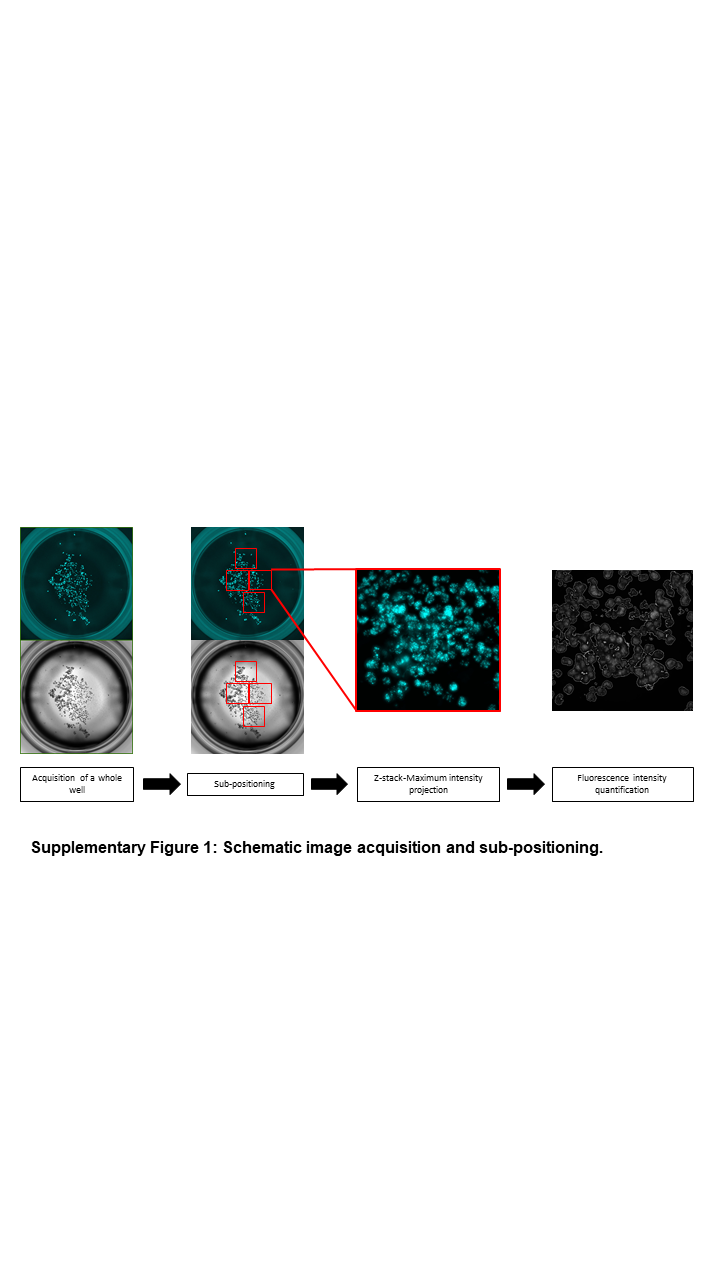

Supplement: Supplementary file 3 [file Image1.tif]
